# Cat Scratch Disease Presenting as Breast Cancer: A Report of an Unusual Case

**DOI:** 10.1155/2013/507504

**Published:** 2013-03-13

**Authors:** Carlo Iannace, Domenico Lo Conte, Lorenzo Di Libero, Antonio Varricchio, Antonio Testa, Raffaella Vigorito, Giuliano Gagliardi, Maria Lepore, Francesco Caracciolo

**Affiliations:** ^1^San Giuseppe Moscati Hospital of Avellino, General Surgery and Breast Unit, Italy; ^2^Federico II Universitary Hospital of Naples, General Surgery, Italy; ^3^Victor Babes University of Medicine and Pharmacy, Timisoara, Romania; ^4^Santa Maria alle Scotte University Hospital of Siena, Hematology, Italy; ^5^San Giuseppe Moscati Hospital of Avellino, Radiology, Italy; ^6^San Giuseppe Moscati Hospital of Avellino, Pathology, Italy

## Abstract

Benign lymphoreticulosis (cat scratch disease, CSD) may have a clinical course that varies from the most common lymphadenitis localized in the site of inoculation, preceded by the typical “primary lesion,” to a context of severe systemic involvement. Among these uncommon clinical aspects, there is mammarian granulomatous lymphadenitis which may appear as a mastitis or a solitary intraparenchymal mass, giving the impression of a breast tumor. In these cases, intensive clinical, instrumental, and laboratory investigations are necessary to exclude malignancy. Because of its rarity, in equivocal cases, it is reasonable to use surgical excision for accurate histological examination. We report a case of CSD of the breast in a 59-year-old woman, analyzing the clinical, histopathological, and instrumental appearance and also performing a literature review.

## 1. Introduction

Benign lymphoreticulosis (cat scratch disease, CSD) is a zoonotic disease from Bartonella henselae, which usually manifests as a localized granulomatous lymphadenopathy near the site of inoculation after a cat scratch or other pet's one. In a minority of cases, it can have a severe systemic involvement with splenitis, mediastinitis, and encephalitis [1–3]. 

The mammarian localization is very uncommon, sporadic reports are given in the literature [4–12]. During the clinical and instrumental investigations, it may appear as a solitary intraglandular mass mimicking inflammatory breast cancer.

We report an unusual case of CSD with breast localization in a 59-year-old woman, describing the clinical, histopathological, and instrumental appearance, also performing a literature review.

## 2. Case Report

A 59-year-old white woman, without having significant medical or surgical history, having familiarity with breast cancer, and not undergoing regular mammographic screening, shows the occurrence of palpable mass in the upper outer quadrant of her right breast accompanied by pain fever. 

On physical examination, we detect the presence of a nodule of the right upper outer quadrant. This nodule has a maximum diameter of about 2 cm, it is painless, fixed on the deep layers, and hard. Moreover, palpable lymph nodes in the ipsilateral axilla and the presence of a whitish skin lesion, slightly elevated (diameter 0,5 cm) in the intermammillar region 2 cm below the angle of Luis, are observed.

**Figure 1:**
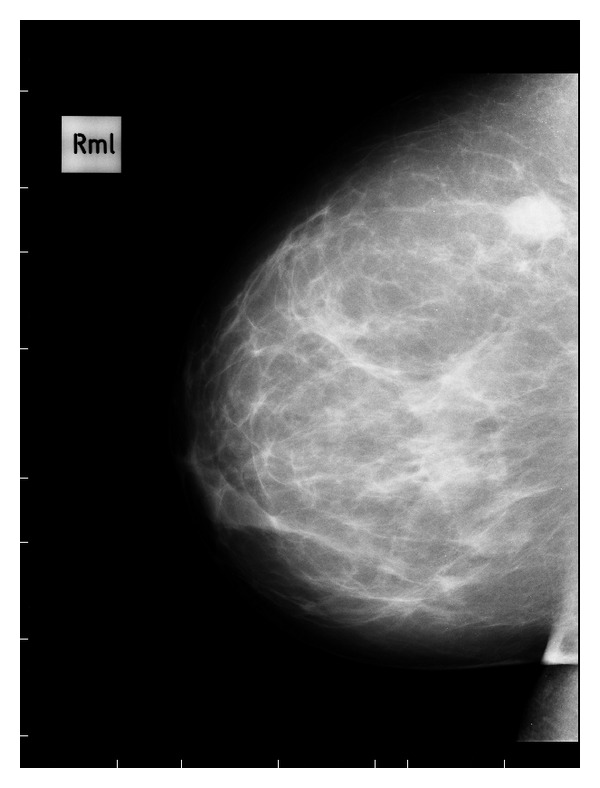
Mid-lateral mammogram of the right breast shown in the upper outer quadrant in a radiopaque mass with margins irregular and speculated.

The mammogram showed a radiopaque nodule about 20 mm, with irregular and spiculated margins (Figure 1). 

**Figure 2:**
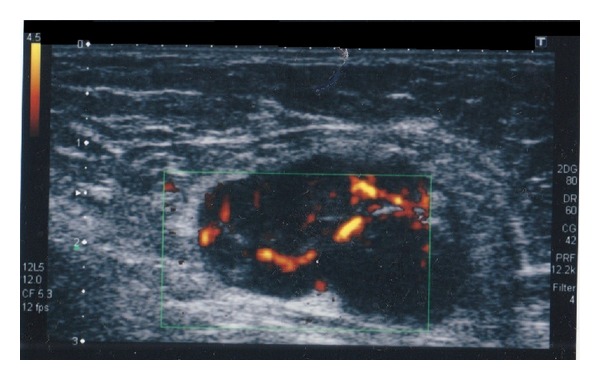
Ultrasonography of the left axillary shows an enlarged lymph node of about 35 mm with vascular poles at color-Doppler.

**Figure 3:**
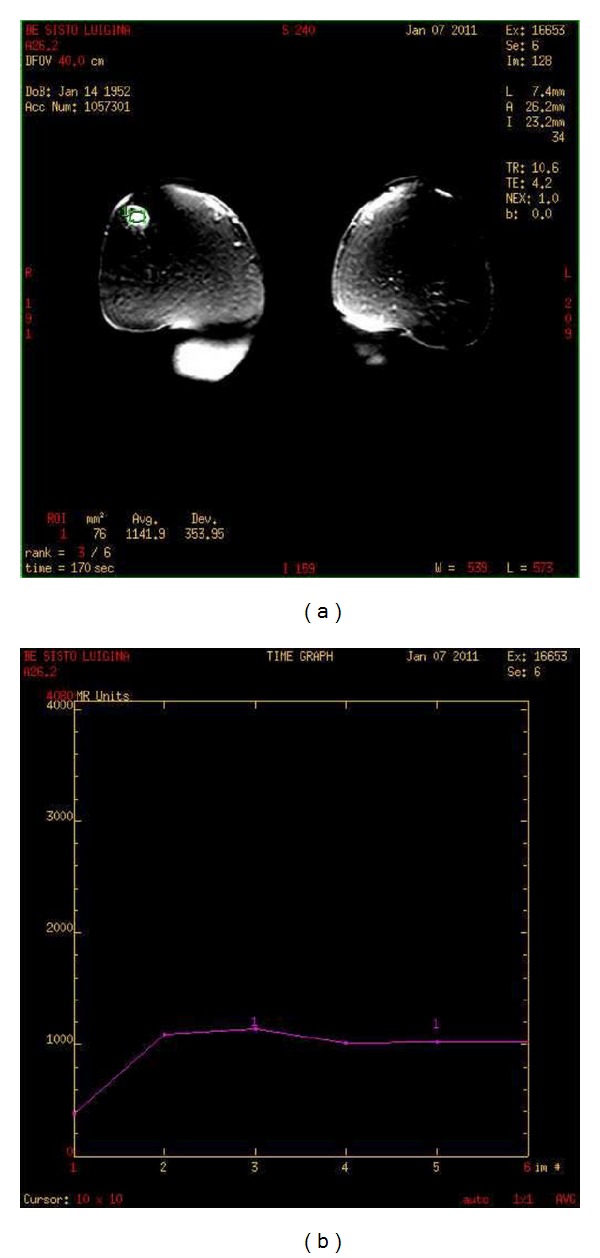
ROI on the lesion and curve *I*/*T* type 3 (malignant).

On ultrasonography mass appears a hypoechoic with irregular margins, with dimensions of 12,5 × 12,5 mm, highly suggestive of heteroplastic lesion; furthermore, enlarged lymph nodes in the contralateral axilla (diameter 35 mm) with vascular poles are identified (Figure 2). On MRI, the lesion shows thin marginal spiculature and an early marked increase in the signal after contrast with a peak at the second minute, marking itself compatible with focality type 4 BIRADS (BIRADS was developed by the American College of Radiologists as a standard of comparison for rating mammograms and breast ultrasound images. It sets up a classification for the level of suspicion (LOS)—the possibility of breast cancer. BIRADS 4 means that findings do not definitely look like cancer but could be cancer. The radiologist is concerned enough to recommend a biopsy) (Figures 3(a) and 3(b)). 

It runs fine needle aspiration of bilateral axillary lymphadenopathy; in both cytology smears, it did not detect neoplastic epithelial cells. Laboratory investigations of the values of CEA and CA 15–3 are normal, as well as inflammatory markers and white cell count.

 Given the clinical and instrumental impression of malignancy, we proceed to a complete surgical excision of the mass with quadrantectomy, ipsilateral axillary dissection, and a biopsy of contralateral axillary enlarged lymph nodes with an extemporaneous exam, the skin lesion is also removed. 

**Figure 4:**
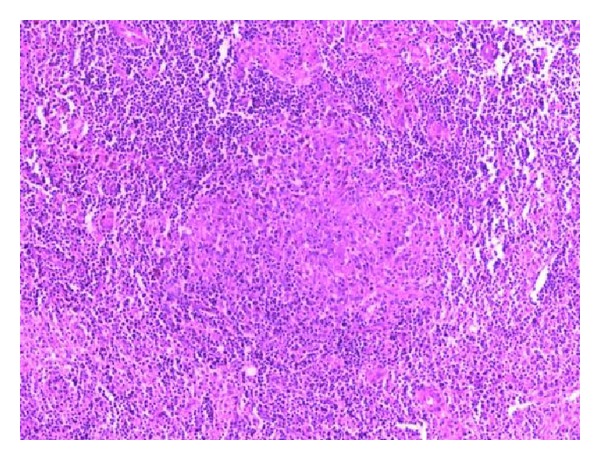
Characteristic suppurative granulomas and stellate microabscess formation (H and E, ×20).

Histologically, the breast lesion is composed of a granulomatous and necrotizing inflammation (Figure 4). The granulomas are composed of elements such as histiocytes with occasional multinucleated giant cells, extensive inflammatory infiltration externally, and extensive areas of confluent necrosis. A similar picture is observed on both axillary lymph nodes and skin lesion. The colors and culture test for fungi, typical and atypical mycobacteria, and immunohistochemistry for toxoplasmosis are all negative. The Warthin-Starry silver staining, specific for Barthonella henselae is negative.

At a later and a more accurate anamnestic investigation, the patient reveals that he had repeated contact with domestic animals and that he had observed, prior to breast pain, the appearance of a lesion such as an erythematous papule on the chest wall and on the site of a skin lesion removed surgically. After five weeks from surgery, the specific serological test is positive for Bartonella henselae.

Upon this positivity, the patient is subjected to azithromycin for three weeks. After five weeks, posttreatment shows a subcutaneous swelling on the right arm that on ultrasound has mixed echogenicity, similar to abscess. This swelling subsides spontaneously; the chest TC shows no active pleuropulmonary disease, and abdominal ultrasound excludes hepatosplenic involvement. The patient underwent clinical and instrumental followup every six months.

## 3. Discussion

In the classic form of CSD, typical of immunocompetent individuals, after 3–10 days from cutaneous inoculation it develops the characteristic of “primary lesion”, that progresses through the stages of vesicle and erythematous papule, resolving within 1–3 weeks. This is associated with lymphadenopathy in one or more of the injected satellite stations. 

The most frequently involved lymph nodes are the anterior cervical, preauricular, supraclavicular, axillary, epitrochlear, inguinal, and femoral [1–3, 8]. 

In immunocompromised patients, the infection may progress to a systemic form, with involvement of internal organs. A microscopic evaluation of the involved lymph nodes reveals the presence of fibrosis of the capsule and subcapsular sinus, follicular hyperplasia, hypertrophy of the germinal centers, and a typical granulomatous reaction with central necrosis in 3 of 4 patients [6].

Our patient showed a picture of a lesion pathologically similar. The diagnosis is made in presence of three of the five following criteria: (1) a history of contact with animals and finding a scratch or a cutaneous “primary lesion”; (2) the presence of regional lymphadenopathy; (3) the exclusion of other possible causes of lymphadenopathy by serological and/or culture test; (4) a positive intradermal test with specific antigen; (5) the typical histopathological changes in lymph nodes examined. 

If typical pleomorphic bacilli are demonstrated by the Warthin-Starry stain, the diagnosis is made also with the positivity of only one of the criteria outlined earlier [2]. Using this staining of formalin-fixed tissues, you can increase the visibility of microorganism through the deposition of silver [13, 14]. However, in early lesions they may not appear [9].

In these cases, the search by PCR of microbial DNA may still be positive [15]. Usually, antimicrobial therapy is not required, because the process tends to limit itself. However, a positive response to different antimicrobials is reported in the literature, including azithromycin, which is considered the first-choice drug [9, 16]. The CSD of the breast is really an unusual clinical entity. 

Lefkowitz and Wear in 1989 reported the first four cases, being able to identify the characteristic bacilli by Warthin-Starry silver staining [4].

Chess and colleagues are the first to describe the sample obtained by FNAC in the CSD of the breast. Their sample is negative on Warthin-Starry staining; however, they make a diagnosis of CSD by a history of cat scratch and a positive intradermal test [5]. Its important diagnostic role is reported in the literature, especially in patients with peripheral lymphadenopathy and after surgery in patients with breast lesions. The pus aspirated from lymph nodes is used for intradermal injection. In positive cases, the injection of antigen is followed by the appearance of erythema with central swelling at the site of inoculation within 24–48 h. However, this test is not widely practiced for the lack of a standardized preparation [7].

Our report illustrates a truly rare case of CSD localized in the breast, whose clinical and instrumental aspect mimicked that of a breast cancer. 

In particular, the results of MRI with a contrast agent has shown a curve *I*/*T* (Figure 3(b)) with rapid wash-in, plateau and the presence of wash-out, then typo 3 (typical of malignant lesions), a compatible morphological appearance, therefore, a score 4 to the classification of BIRADS.

This induced us not to consider the need for a fine needle aspiration of the lesion in the right upper outer quadrant, because we have already considered malignant, but to perform a fine needle aspiration of multiple and bilateral axillary lymphadenopathy. This was negative for neoplastic cells. We proceeded to surgery as described earlier and not to aspirate the intraparenchymal lesion, whose negativity for tumor cells, in disagreement with the clinical and instrumental aspect of malignancy, would induced us, in any way, to take an aggressive approach, such as that of surgical treatment.

## 4. Conclusion

CSD with mammarian granulomatous lymphadenitis is a very rare event, so it is difficult to consider it in the differential diagnosis of breast nodules. In case of clinical and instrumental doubt, it is reasonable to use a surgical approach to obtain an accurate histologic diagnosis of the lesion's nature.
